# Immunization against Viral Hepatitis B: Lessons Learnt from Kingdom of Cambodia

**DOI:** 10.5005/jp-journals-10018-1210

**Published:** 2017-05-05

**Authors:** Bun Sreng, Ork Vichit, Yuong Vuthikol, Chum Aun, Chham Samnang

**Affiliations:** 1 Department of Communicable Disease Control, Ministry of Health, Phnom Penh, Cambodia; 2 Department of National Immunization Programme, National Centre for Maternal and Child Health, Phnom Penh, Cambodia; 3 UNICEF, Phnom Penh, Cambodia; 4 World Health Organization, Phnom Penh, Cambodia

**Keywords:** Cambodia, Hepatitis B virus, Immunization.

## Abstract

An account of immunization against hepatitis B virus in Cambodia is given.

**How to cite this article:** Sreng B, Vichit O, Vuthikol Y, Aun C, Samnang C. Immunization against Viral Hepatitis B: Lessons Learnt from Kingdom of Cambodia. Euroasian J Hepato-Gastroenterol 2017;7(1):43-47.

## INTRODUCTION

It was estimated that 400 million people were infected with viral hepatitis B, and 6 and about 10 million are newly infected every year.^1^ The World Health Organization (WHO) projected that 2 to 3 million deaths can be avoided every year through immunization.^2^ Chronic hepatitis B virus (HBV) infection was the commonest cause of hepatocellular carcinoma.^3^ Viral hepatitis was mostly transmitted through the mother-to-child mode.^4^ The risk of transmitting HBV from the mothers to the newborns accounted approximately 12% when mothers have a high hepatitis B viral load.^5^ The prevalence of HBV varied by geographical areas, the places of the delivery, the residence, the affluence, and the ethnicity.^5^

Different interventions are observed in different countries. Tables 1 and 2 describe those interventions.^3,6^

The immunization against HBV is considered the most effective way to control and prevent this major life-threatening communicable disease. It can prevent the infection and development of chronic disease and liver cancer due to hepatitis B (95% effectiveness).^2^

**Table 1:** **Table 1:** Interventions to prevent mother-to-child transmission (PMTCT) of HBV

• Universal screening of hepatitis B among pregnant women	
• Hepatitis e antigen (HBeAg)	
• HBV DNA level in HBV-infected mothers	
• Series of hepatitis B vaccines (active immunization)	
• HBIG (passive immunization)	
• Antiretroviral (tenofovir) at 3rd trimester	
• Cesarean section	

**Table 2:** **Table 2:** Schedule of the viral hepatitis B vaccination in Kingdom of Cambodia

*Within 24 hours (up to 7 days)*		*6 weeks*		*10 weeks*		*14 weeks*	
Monovalent		DPT-HepB-Hib		DPT-HepB-Hib		DPT-HepB-Hib	
hepatitis B		(pentavalent)					

In 1992, the WHO set a goal that all member states shall introduce hepatitis B vaccine into the Universal Childhood Hepatitis B vaccination by 1997. It set up the elimination targets of less than 2% by 2012 and of less than 1% by year 2017. By 2005, the vaccination coverage shall be at least 80% of birth cohorts in every district receiving three doses of hepatitis B vaccine.^7-9^

The WHO categorized Cambodia as one of the countries with high prevalence of viral hepatitis B. Cambodia introduced the hepatitis B vaccine in 2000, and a pilot project was conducted in Kampong Chhnang Province. In 2002, it started to scale up nationwide.

The National Immunization Programme (NIP) developed a Comprehensive Multi-Year Strategic Plan, 2016 to 2020, covering five areas namely service delivery, behavioral communication, cold chain management, vaccine preventable disease surveillance, and program management to reach the immunization’s set targets.^10^

The objectives of this study are as follows:

- Review key interventions by the NIP
- To profile the achievements of the NIP
- Conduct strengths, weaknesses, opportunities, threats (SWOT) related to implementation of the hepatitis B vaccine
- Provide recommendations to improve the hepatitis B vaccine coverage.

## MATERIALS AND METHODS

- Review policies, strategic plans, guidelines, protocols, standard operational procedures (SOPs),
- Review NIP’s reports, monitoring, evaluations, and publications related to NIP
  – Vaccination coverage for hepatitis B
  – Implementation of strategic plans
  – Health system to support NIP
  – Partnership
  – Budget to support NIP (procurement of vaccines, program’s management)
- Interview key stakeholders including NIP’s management at national (NIP, Financing, CMS), subnational (PHDO, ODO, RH, HC), and community (VHSG), partners (WHO, United Nations International Children’s Emergency Fund [UNICEF], Global Alliance for Vaccination Initiative [GAVI], RACHA)
- Literature review

## RESULTS

### Activities Related to Key Strategic Areas 2016 to 2020

There are a number of key policies, strategic plans, guidelines, and SOPs, which were developed by the NIP. Those listed are National Immunization Committee, National Policy 2012, Strategic Plan 2016 to 2020 (2016), National Strategy for the Introduction of Hepatitis B Vaccine, User Guide for Refrigerator Temperature Monitoring Using Fridge-Tag 2014, and Operation Guidelines Surveillance of Adverse Events Following Immunization (AEFI) 2015.^11^

Currently, the NIP’s vaccination calendar is comprised of nine types of vaccines, namely Bacillus Calmette Guerin (BCG), hepatitis B, oral polio vaccine, diphtheria-polio-tetanus-hepatitis B-hemophilus influenza B (DPT-HepB-Hib), pneumococcal conjugate vaccine (PCV13), inactivated polio vaccine, measles/ rubella at 9 months (MR9), measles/rubella at 18 months (MR18), and Japanese encephalitis.^10^

All levels of the health system (national, provincial, district, health centers) are required to develop planning including routine and supplementary immunization activities. The routine immunization consists of fixed and outreach sessions. Fixed sites include health centers and hospitals. The maternity ward provides only HepB birthdose and BCG.^10^

The government plans to increase the budget’s contribution from 31% in 2016 up to 43% in 2020. The second major funding source is the GAVI. The rest are UNICEF and WHO.^11^

### Performance of the NIP Based on Key Indicators

**Graph 1:**
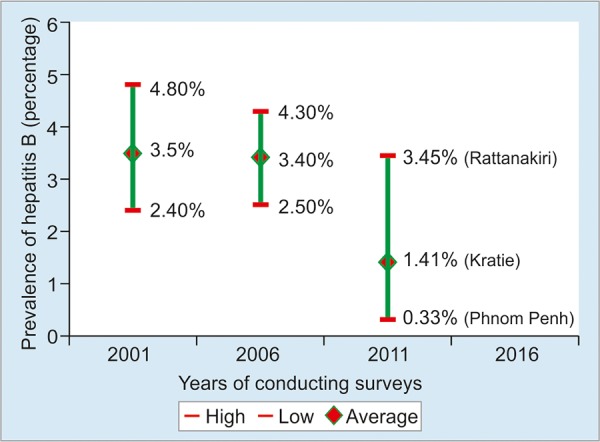
Prevalence of hepatitis B infection in children aged 5 years, 2001, 2006, 2011, and 2016 Sources: Seroprevalence surveys 2001, 2006, 2011

The prevalence of hepatitis B vaccine has decreased slightly from year-to-year and from one province to another depending on different factors (Graph 1). The prevalence was 3.5% (2.40-4.80%) in 2001 and 3.40% (2.50-4.30%) in 2006 and 1.41% in the Kratie province in 2011 (3.45% in Rattanakiri province and 0.33% in Phnom Penh). The seroprevalence survey 2016 will be conducted in early 2017.^12-14^

**Graph 2:**
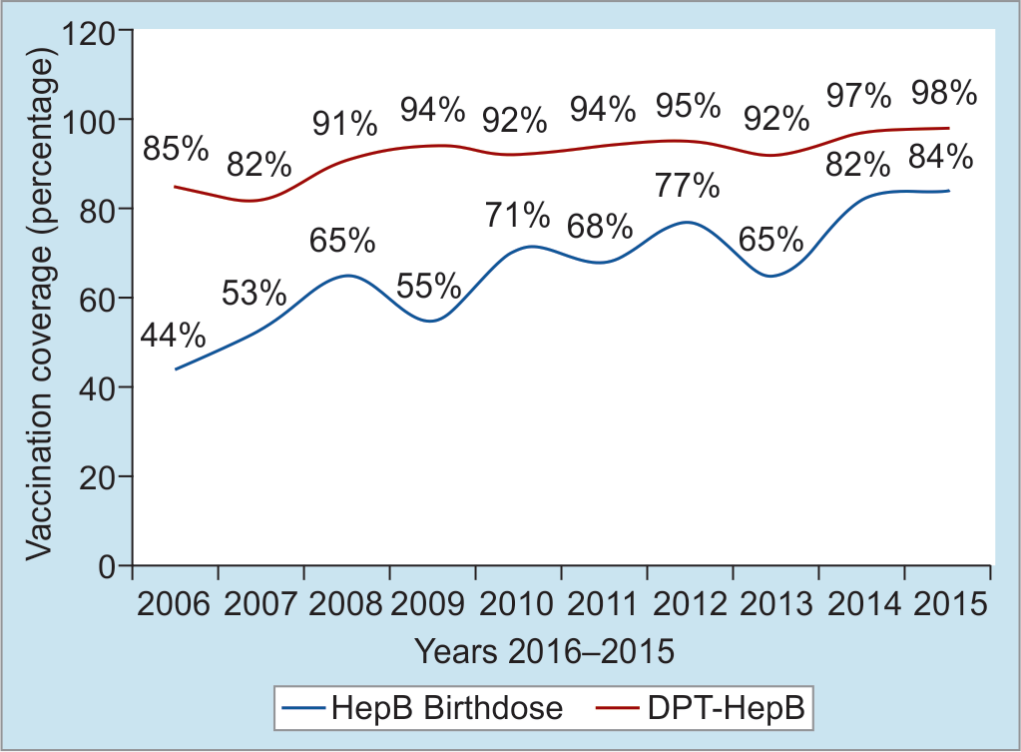
The coverage of hepatitis B vaccine in Kingdom of Cambodia, 2006 to 2015 Sources: National immunization programme (Cambodia)

The coverage of hepatitis B vaccine has increased constantly and is very high for both hepatitis B vaccine birth doses (BD) and DPT-HepB (Graph 2). The coverage of the hepatitis B birth dose doubled from 44.0% in 2006 up to 84.0% in 2015; DPT-HepB from 85% in 2006 up to 98.0% in 2015.^15^

**Graph 3:**
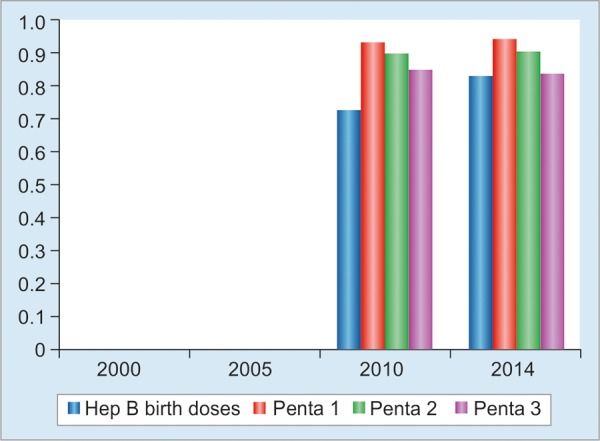
The coverage of hepatitis B vaccines, 2010 and 2014 Sources: Cambodia demographic health surveys 2010 and 2014

The Cambodian Demographic Health Surveys conducted in 2010 and 2014 showed similar figures with the reported vaccination coverage by the Health Information Management System of the Ministry of Health of Cambodia as well (Graph 3). It was observed that there was a slightly decreasing trend between each pentavalent vaccine 1, 2, and 3.^16^

**Graph 4:**
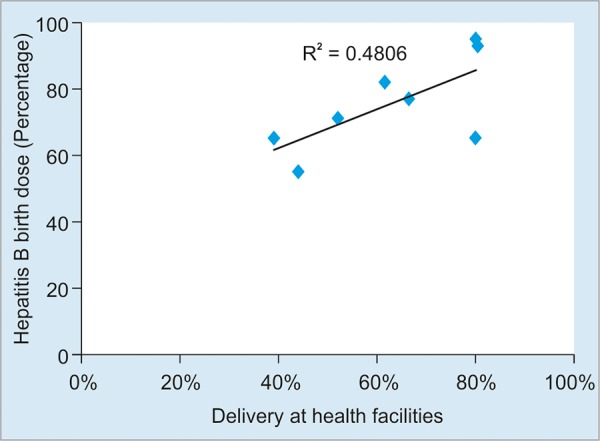
The correlation between delivery at health facilities and hepatitis B birthdose (BD) coverage, 2008 to 2015

We observed that higher was the proportion of pregnant women who delivered at the health facilities (Graph 4).^17^

### Results of SWOT Based on NIP Strategic Plan 2016 to 2020

The SWOT analysis has been considered as a good tool for a program’s review. The tool was used to review the performance of the NIP at national and subnational levels based on its current policies and strategic areas. The summaries of SWOT analysis related to service delivery, behavioral communication, cold chain management, vaccine preventable disease surveillance, and program management are listed in Table 3.

## DISCUSSION

### Prevalence and Coverage of Hepatitis B

Cambodia is one of the countries with the highest hepatitis B prevalence among the general public in the world. It was observed that the prevalence of hepatitis B in 5-year-old children decreased slightly from 2005 through 2011. This was due to the introduction of the HepB vaccine in 2001; its coverage in 2006 was also low at around 40% for HepB birthdose and 85% for pentavalent (Graph 2 and 3). In an early 2017 survey, it is expected that this prevalence might be below 1% because the birthdose coverage almost doubled in 2010 (71%) compared with 2006.

The coverage of hepatitis B vaccine birthdose and three doses of pentavalent at 6, 10, and 14 weeks was very high in Cambodia in 2015. This may result in the further reduction of the HepB prevalence among 5-year-old children.

However, the seroconversion has been found low also in some countries. In this particular regard, in some countries with high burden of viral hepatitis B, universal blood screening including hepatitis B antigen, HBe antigen, and HBV deoxyribonucleic acid (DNA) is done, and corresponding actions apart from routine HepB vaccine are composed of hepatitis B immunoglobulin (HBIG), antiretroviral, and cesarean section. Those interventions are provided to increase its effectiveness. Additional measures include the universal testing of pregnant mothers for hepatitis B, hepatitis e antigen, and viral DNA among those who are infected with hepatitis B, where the health infrastructure is capable of performing testing.

### Service Delivery, Cold Chain, and Program Management

Fixed sites immunization has improved remarkably reflecting the awareness of the mothers about the importance of having their children vaccinated and the increase of deliveries at the health facilities.

Outreach activities have played an important role in reaching newborns in high-risk areas. Like other countries, cold chain has been an issue in some countries, especially, in very remote areas and to address this particular issue, the out-of-cold chain approach has been applied in some places of some countries. The policy of the Ministry of Health has made availability of HepB vaccine in very remote communities provided by the community health workers (China).^18^

**Table 3:** **Table 3:** The results of the analysis using SWOTs

*Strategic areas*		*Strengths*		*Weaknesses*		*Opportunities*		*Threats*	
Service delivery		• Management structure at ALL levels – Fixed sites (69%) – Outreach (31%) focusing on high-risk villages (n = 1,834) • Special supply for campaign (6 week long period): 3 groups of provinces and at different dates		• Quality of staff and interventions • Overreporting • Delivery at home and delayed timely birth dose (TBD) > 24 hours, especially in remote areas • Limited information about vaccination from private sectors • Limited budget for outreach activities and infrequent (2-3 months)		• Antenatal Care 4 (ANC 4) and postnatal care 1 (PNC 1)? • Health facility deliveries • Integrated management of childhood illness • School enrollment (?) • Raising awareness (nongovernmental organizations)		• Demographic challenges: Residence (urban *vs* rural), provinces, mother’s education, wealth quantiles • Mobile and migrant populations • High-risk villages • Competition with other health priorities	
Cold chain management		• Prequalified refrigerators and vaccines • Updated inventory and replacement plan of cold chain • Introduction of Fridge-Tag 2 to monitor temperature • Assessment and improved plan of Effective Vaccination Management (EVM) every 3 years (2012, 2015)		• Limited cold chain maintenance system • Limited number of cold chain technicians (n = 01) at the national level for about 1,700 refrigerators nationwide • No systematic for having preventive maintenance system • Low scoring of EVM		• Plan to assess cold chain maintenance system • GAVI’s funding for cold chain replacement • Operational budget from government for HCs • Power and gas supply from government		• Staff turnover (retired, moved away) responsible for cold chain maintenance	
Behavioral communication		• Draft Communication Strategic Plan 2015 (WHO) • Provide education to the public during outreach, fixed sites and campaign (TV, radio, posters, leaflets)		• Weak community awareness and demand including. No recent KAP survey on immunization (EPI staff, public)		• Improved socioeconomic status of the population • Increased coverage of the media, especially social media		• Minimal (increase in demand for vaccination) for high-risk groups	
Vaccine preventable disease surveillance		• Periodical seroprevalence surveys (2001, 2006, 2011, 2016) • Health management systems (national and provincial hospitals) for hepatitis B and hepatitis C		• Hepatitis B not included in surveillance policy for NIP vaccine preventable diseases • Limited testing capacity for hepatitis B at subnational level		• Periodical seroprevalence using external funding • PMTCT		• External funding for periodical seroprevalence survey of hepatitis B • Limited diagnostic kits supplied by government	
Program management		• Annual vaccine forecast (2017-2021) • EPI staff at ALL levels trained		• EPI staff: Shortage, burden of work, high turn-over, with limited quality at ALL levels • Work plan’s implementation, shared technical responsibilities at each levels		• GAVI’s funding to support capacity building		• External funding sources for capacity building	

EPI: Expanded programme of immunization

### Behavioral Communication

The NIP shall continue to raise awareness about the importance of the immunization, especially with focus on promoting health facility delivery, and also on the birth dose and pentavalent 1, 2, and 3 administration using current strategy, particularly, for those who live in high risk areas.

## RECOMMENDATIONS

The NIP should maintain current implementation of the activities according to its policies and strategic plans. Both fixed sites and outreach activities including high-risk communities should be maintained. The capacity building shall be provided, especially, to those who are newly assigned. The cold chain shall be maintained and strengthened. Government’s funding shall be secured and increased when the external funding has decreased. Vaccination targeting selecting adult groups, especially, the health professionals shall be considered.^19^

The NIP should consider additional measures including testing pregnant mothers for HepB antigen, HBe antigen, and HBV DNA for those who are able to afford to pay. Based on those measures, it can consider using HepB HBIG and antiretroviral.^20-23^

## CONCLUSION

The NIP has done a remarkable job and the HepB birth-doses and three pentavalents have been very high since 2010. The vaccination coverage was very high, especially, the birthdose. Additional measures shall be considered by revising the current national policy and strategic plan as mentioned in the recommendations.
